# Primitive Neuroectodermal Tumor and Wegener's Granulomatosis of the Kidney: A Curious Combination of Two Rare Entities

**DOI:** 10.1155/2017/1750694

**Published:** 2017-08-01

**Authors:** Rugvedita Parakh, Satyajeet Parakh, Maria Tretiakova

**Affiliations:** ^1^Department of Pathology and Laboratory Medicine, University of Washington, Seattle, WA, USA; ^2^IP Spring, Chicago, IL, USA; ^3^Department of Pathology, University of Washington, Seattle, WA, USA

## Abstract

Wegener's granulomatosis (WG) is characterized by necrotizing polyangiitis involving the respiratory tract and kidneys. It causes segmental necrotizing glomerulonephritis in the kidneys. In rare cases, a renal pseudotumor may be seen because of the granulomatous process. Association of WG with renal malignancy, however, is very uncommon. We report a case of a patient who presented several years after being treated for WG with malignant hypertension and an infiltrating mass in the right kidney. The histopathology of radical nephrectomy specimen showed presence of primitive neuroectodermal tumor (PNET). Association of renal cell carcinoma (RCC) with WG has been documented in a few cases, but PNET in such circumstances has not been reported. Long-term immunosuppressive treatment is a known risk factor in the development of malignancies, so it is proposed that the occurrence of RCC in WG may have been a side effect of cyclophosphamide treatment. It is not clear whether the same mechanism for PNET holds true in the present case. It is important to make a differential diagnosis between true malignancy and pseudotumors in WG as these entities cannot be distinguished based solely on imaging. We suggest a need to routinely screen the WG patients for increased risk of urologic malignancies.

## 1. Introduction

Wegener's granulomatosis (WG) is an antineutrophil cytoplasmic antibody (ANCA) associated systemic necrotizing granulomatous vasculitis of unknown etiology. WG predominantly causes necrotizing granulomatous inflammation of the upper and lower respiratory tract. The kidneys are involved in only 20% of cases at initial presentation; however, WG eventually involves the kidneys in approximately 80% of cases [1]. Although the characteristic manifestation of renal involvement by WG is segmental necrotizing glomerulonephritis with proteinuria that often culminates into a rapidly progressive renal failure, few cases may present with fibroinflammatory renal masses [2]. These pseudotumors are asymptomatic and are usually detected incidentally upon radiology [1]. Renal cell carcinoma (RCC) needs to be ruled out histopathologically in such cases as cooccurrence of RCC with WG, although rare, has been reported [3, 4]. This cooccurrence may be attributed to similar pathogenetic pathways or may be iatrogenic owing to the effect of therapy for WG including immunosuppression [1, 2].

Primitive neuroectodermal tumor (PNET) is a comrade of Ewing's sarcoma family of tumors which is hypothesized to arise from primitive cells of neural crest. It is a highly aggressive tumor that most commonly involves the bone and soft tissue of young adults [5]. Renal PNETs are rare with less than 120 reported cases [6, 7]. The differential diagnosis from other renal malignancies carries important therapeutic considerations [8]. Renal PNET and WG have never been described together, and we do not know whether it's spurious association or if there is some underlying pathogenetic mechanism involved. We report, for the first time, an intriguing case of renal PNET occurring in a young adult female after 7 years of diagnosis of WG.

## 2. Case Presentation

A 30-year-old woman initially presented with high fever and cough in 2007, and, within ten days, developed joint, back, and chest pains, epistaxis, hemoptysis, dyspnea, and a vesicular hemorrhagic rash. She lost 15 kg in one month and was hospitalized with a high fever, 135/110 mmHg blood pressure, and an erythrocyte sedimentation rate of 80 mm/h. A 24-hour urinary protein excretion was measured at 0.9 g/L with microscopic hematuria, and she had a positive serum test for PR-3 levels. The antineutrophil cytoplasmic antibodies (ANCA) were positive on the biopsy which revealed crescentic and necrotizing glomerulonephritis. The chest X-ray showed multiple nodular and patchy shadows throughout both lungs and a prominent left hilum. She was diagnosed with WG and started with pulse doses of glucocorticosteroids after which cyclophosphamide (CYC) was instituted, with tapering of corticosteroid dose. The symptoms soon resolved and remission was achieved.

In the second year of her ongoing treatment, because of the development of anemia, the patient experienced two periods of relapse and, after reinstituting therapy (three pulses of methylprednisolone 500 mg and then two pulses of CYC 1000 mg; after two weeks, oral CYC 100 mg/d therapy was reinstituted), went into remission. The total cumulative dose of CYC was about 150 grams.

**Figure 1:**
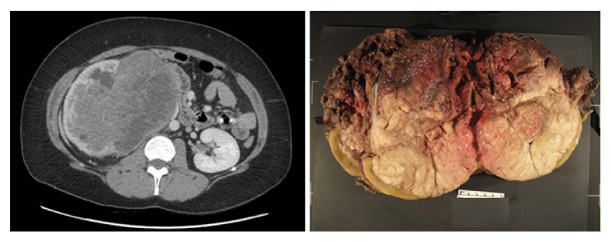
CT imaging shows a heterogeneous partially calcified enhancing solid mass of the right kidney and gross findings show a necrotic and friable soft tissue mass involving the distal ureteric margin and the renal vein.

Seven years later (2014), the patient complained of pain in the right lumbar region. She also had malignant hypertension. She was a nonsmoker with no exposure to occupational carcinogens and a negative familial history for renal malignancies; her body mass index was 28.7 kg/m^2^. She was also noted to have a blood pressure of 170/111 after initiation of oral contraceptive pills. The blood pressure did not subside even after discontinuation of pills or with medical management. A CT scan revealed an infiltrating mass of 17.0 × 16.3 × 12.2 cm in size in the right kidney; the lesion appears confined to the perinephric space. Lesion is noted adjacent to and compressing the right renal artery with anteromedial displacement (Figure 1). Laboratory analysis of blood and urine showed normal values, while the ANCA test was negative, suggesting that her WG was still in remission. Patient was admitted for surgical removal of the tumor identified on the CT scan; after surgical exploration, an open radical nephrectomy was performed. The right kidney and mass weighed 1475 g and measured 17.0 × 13.0 × 10.0 cm. The normal renal parenchyma measured 1.5 cm with a 0.5 cm cortex. There was a large necrotic soft mass invading the hilum measuring up to a maximum dimension of 16.0 cm. Large necrotic and hemorragic areas were noted. The distal ureteric margin and the renal vein were involved; however, no renal capsular involvement was present. Upon further dissection, the tumor was seen abutting the pelvis rather than invading it, although it did appear to obliterate the renal sinus (Figure 1).

**Figure 2:**
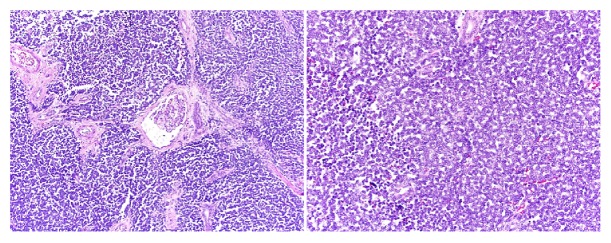
Diffusely infiltrative small blue cell tumor, magnification (100x); round cells with scant cytoplasm and moderate degree of nuclear atypia, magnification (200x).

**Figure 3:**
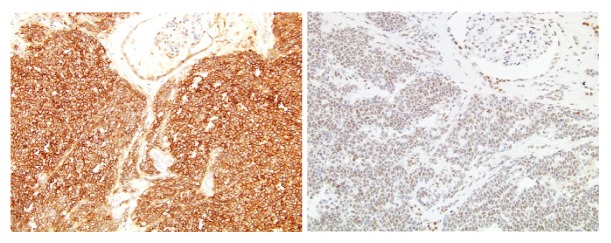
Diffuse and strong membranous positivity for CD99, magnification (200x); diffuse and moderate nuclear positivity for FLI-1, magnification (200x).

**Figure 4:**
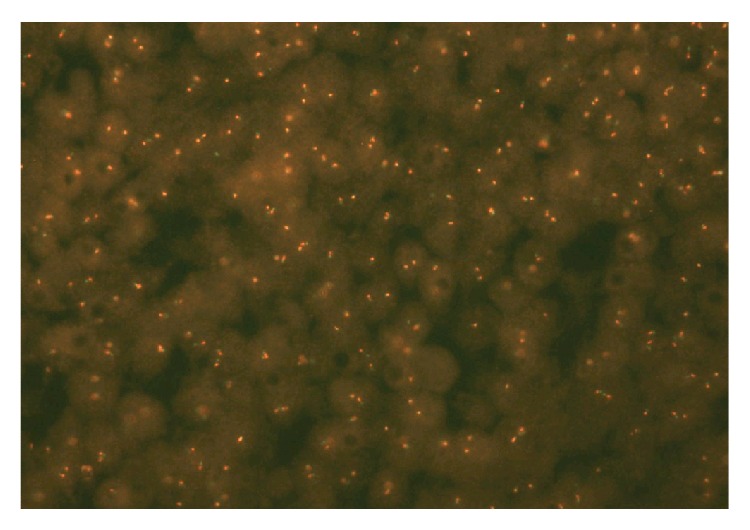
FISH studies: EWS gene rearrangement.

Microscopy showed primitive looking small blue cells widely infiltrating and destroying renal parenchyma and positive surgical margins. In the nonneoplastic kidney parenchyma, there were signs of glomerular sclerosis and vessels showing fibromuscular hyperplasia. Nephroblastoma, lymphoma, sarcomatoid renal cell carcinoma, and rhabdomyosarcoma were brought into the differential diagnostic considerations and were ruled out, and a diagnosis of renal PNET was offered (Figure 2). The histopathologic diagnosis was confirmed by immunohistochemistry with characteristic positivity for CD99 and FLI1 (Figure 3), as well as FISH studies documenting EWS gene rearrangement (Figure 4).

The patients' hypertension was resolved after the surgery. The patient was followed up regularly and decided to transition to palliative care because of her deteriorating condition. One and a half year after the surgery, she presented with malignant biliary obstruction and acute liver failure due to widespread metastasis and succumbed to death in 2016.

## 3. Discussion

Primitive neuroectodermal tumor (PNET) is thought to arise from primitive cells of neural crest. Patients with renal PNET present with nonspecific symptoms such as malaise, fever, flank pain, hematuria, night sweats, and dyspnea. Serum biochemistry values may show elevated LDH, glutamic-oxaloacetic transaminase, glutamic-pyruvic transaminase, and creatinine. In the present case, the patient presented with malignant hypertension, which subsided with renal resection. This is an interesting finding suggesting that PNET may have been the cause of malignant HT. The age of the patient was 37 years, much later than the typical childhood tumors. She received aggressive cyclophosphamide (CYC) therapy, which remains the mainstay therapy for WG.

The current understanding about pathophysiology of WG is a constellation of multiple immune abnormalities that culminate in the overproduction of autoantibodies directed against proteinase 3 (PR3-ANCA). Therefore, immunosuppressive agents such as CYC are commonly used. Patients with WG are at a markedly increased risk of bladder cancer, with absolute risks as high as 10% some years after diagnosis. Increased risk may partly be attributed to CYC and some other factors even before the diagnosis of WG [9]. Long-term oral CYC therapy is associated with substantial urotoxicity in 25% of patients [1], including the development of urothelial carcinoma of the urinary bladder. In another cohort of patients, the estimated incidence of bladder cancer after the first exposure to CYC was 5% at 10 years and 16% at 15 years [10]. It has been reported that treatment with CYC as a single agent or in combination therapy can cause secondary malignancies such as myelodysplasia, acute leukemias, lymphomas, thyroid cancer, and sarcomas [11]. The onset of these secondary malignancies may be delayed up to several years after treatment [12, 13].

The pathophysiology for PNET is driven by the fusion of EWS-FLI1 genes triggering and stimulating oncogenesis. It is not yet known whether the immunosuppressive agents like CYC can facilitate the fusion of the above genes or unmask the underlying mechanism. Interestingly, adult PNET/Ewing sarcomas are usually treated with vincristine, doxorubicin, and CYC with fairly good response and 2-year survival at 80% [14, 15].

Renal cell carcinoma (RCC) development in WG patients undergoing CYC therapy has been also reported; however, a direct association is yet to be determined [3, 16]. The authors proposed that the occurrence of RCC in WG may have been a side effect of CYC treatment. An association of PNET with WG in such circumstances has not been reported. As described above, long-term immunosuppressive treatment is a known risk factor in the development of multiple secondary malignancies. We could postulate a similar pathogenetic association between prior CYC treatment for WG in developing secondary PNET/Ewing sarcoma and increased resistance to immunosuppressive agents. Also, the nephrotoxic effect of CYC treatment is seen in 1/4 of patients and could be an additional contributing factor to poor outcome in our patient. We support our hypothesis based on a study showing enhanced intravascular proliferation, extravasation, and subsequent colony formation of the HT1080 human fibrosarcoma in mouse model after CYC injection [17]. Thus, it is conceivable that prior exposure to immunosuppressive therapy in our patient induced opposite effects of CYC which enhanced critical steps in PNET pathogenesis.

We did not perform any molecular studies on the biopsy because these studies are not needed to establish WG diagnosis as compared to the PNET, in which case it would be important. Therefore, we are not able to establish if there is a molecular or genetic aberration alluding to WG and PNET association. Could the immunosuppression evoke the underlying malignant tumorigenesis mechanism? Is the occurrence treatment related, or do these entities share a common pathogenetic pathway, or it was simply poor old bad luck? It remains an open question.

A systematic search in multiple databases (e.g., PubMed and ScienceDirect) was performed for articles on WG and Ewing sarcoma family tumors in the kidney. To date, no association between WG and PNET has been reported.

## 4. Conclusion

We recommend careful search and surveillance of the patient with WG for various tumor associations early in the course of the disease. While working up for malignant HT in a young female with WG, its probable association with PNET should also be considered. Differential diagnosis is not difficult with newer IHC markers and awareness, but the clinical and radiological presentation may pose challenges. We also suggest the need to routinely screen the WG patients in view of the increased risk of urologic malignancies. Therefore, an early pathology opinion should be sought. We suggest further studies to establish various neoplasms associated with WG and role of immunosuppressive therapy especially in kidney.
